# Improving Male Partner Involvement in HIV-Positive Women's Care Through Behavioral Change Interventions in Malawi (WeMen Study): A Prospective, Controlled Before-and-After Study

**DOI:** 10.3389/fpubh.2022.864489

**Published:** 2022-07-08

**Authors:** Isotta Triulzi, Fausto Ciccacci, Ilaria Palla, Bryan Mthiko, Darlington Thole, Maria Cristina Marazzi, Leonardo Palombi, Giuseppe Turchetti, Stefano Orlando

**Affiliations:** ^1^Institute of Management, Scuola Superiore Sant'Anna, Pisa, Italy; ^2^Unicamillus, Saint Camillus International University of Health Sciences, Rome, Italy; ^3^DREAM Programme, Community of Sant'Egidio, Balaka, Malawi; ^4^Human Sciences, Libera Università degli Studi Maria Ss Assunta di Roma, Rome, Italy; ^5^Department of Biomedicine, University of Tor Vergata, Rome, Italy

**Keywords:** men's role, AIDS, HIV, intervention study, health education, gender, Malawi, health-related behavior

## Abstract

Several strategies and interventions have been implemented to improve male partner involvement (MI) in Sub-Saharan Africa, but evidence on successful interventions is scarce. This controlled before-and-after intervention study aims to evaluate the impact of three interventions on male partners' involvement in HIV+ women's care in Malawi. We piloted these three interventions: the organization of a special day for men, the deployment of male champions in communities to increase awareness on MI, and the delivery of an incentive (food package) for couples attending the facility. We observed a significant increase in the number of women accompanied by their partners (from 48.5 to 81.4%) and the number of women feeling safe at home (from 63.5 to 95.2%) after the special day intervention. This outcome increased after the deployment of male champions in communities (from 44.0 to 75.0%). No significant improvement was observed in the site where we delivered the incentive to couples. Our findings showed that the special day for men and the use of male champions might effectively increase the male involvement in the health of their female partners.

## Introduction

Male involvement (MI) is a critical issue, especially in maternal and child health (MCH) and in the Prevention of Mother to Child Transmission (PMTCT) of HIV in Sub-Saharan Africa (SSA). In this continent, men plays an relevant role in the decision-making process within the household and this often impacts women's access to healthcare, contraception, food, and work, and, as a consequence, pregnancy outcomes (1–4). Low male involvement can impact on treatment refusal, delayed enrollment, and dropout of pregnant and breastfeeding women (5). Previous literature suggests that male partner support is expressed by providing transport to the clinic, reminding the clinical visits or the medications, and accompaniment to appointments (6–9). Previous studies and reports have found several definitions and attempt to develop a composite index of male support/involvement (10–12), but a unanimous and well-accepted measure has not been found in the literature.

Qualitative studies suggest that women are not supported enough by their male partners in many aspects; some of the barriers reported are access to transport to the clinic, lack of accompaniment to appointments, sometimes men are even reported to hinder female access to the clinic and female retention in care itself (13–16). Gender norms and roles significantly limit women's ability to access healthcare independently of male support (17) since men are often the decision-makers and gatekeepers for the family (18). Potential harms associated with increasing male involvement should be carefully considered to engage them. Increasing men's power over a traditional women's sphere might mean encroaching upon women's domain and may change the hoitausehold's dynamic and the relationship between partners, exacerbate gender asymmetry, and reinforce the patriarchy (10, 19).

In 2020, an estimated 990,000 Malawians were living with HIV, and 12,000 Malawians died from AIDS-related illnesses in the same year. More than 98% of pregnant women were tested for HIV, and more than 95% of those testing positive were on treatment (20). In Option B+ programs, scarce adherence, retention and loss-to-follow-up (LTFU) are still serious issues and keeping patients on long-term therapy is a critical element of HIV care. Two larger studies conducted in Malawi showed that 70% of the 29,313 women who started Antiretroviral Therapy (ART) under Option B+ by June 2012 were still in care 3 years later, and about 30% of the retained women did not adequately adhere during the first 2 years of ART (21, 22). Previous studies extensively describe barriers to adherence (23, 24); among these barriers, male partner support is one critical factor affecting adherence and retention to therapy (13, 14, 16, 25–27).

In Malawi, male involvement in MCH and PMTCT ranges from 3.2 to 74% (28). One study run from January 2004 to December 2006 at Mwanza district hospital reported that only 63 (13.7%) out of 467 HIV-positive women enrolled in PMTCT attended the clinic with their partner (29). A second observational study found that 10.7% of women attended with their partners in Bwaila Hospital in Lilongwe in 2009 (30). A third unblinded randomized controlled trial (RCT) comparing the adoption of the invitation slip vs. the use of invitation slip plus tracing reported that 74 vs. 52% of couples presented to the clinic, respectively (31). Another RCT study showed that among 462 women (June and December 2013), 230 received an invitation card, and of these, 65 (28%) came accompanied. Of the 232 women in the standard of care group, 44 (18%) arrived accompanied (32). A recent observational study showed that a relatively higher portion of HIV-positive women was accompanied by their male partners (64.1%) when the healthcare workers provided an invitation card to women (33).

Similar data are reported in other SSA countries: a Ugandan study reported a high prevalence (74%) of low male involvement, and only 5% of men escorted their partners to the Antenatal Care (ANC) clinic (27). In Kenya, the level of MI amounts to 36% (25) and 53.5% in Tanzania (26).

Several health interventions and strategies have been implemented in Sub-Saharan Africa and Malawi. However, most of these programs have not shown success. Few studies and with variegate study design were conducted as shown by a recent systematic review (34). A previous review shows that psychosocial interventions and complex community interventions increase male partner involvement compared to the invitation letter (35).

Our research aims to assess the impact of three different interventions on male partners' involvement in HIV+ women's care in Malawi (36).

## Materials and Methods

We conducted a controlled before-and-after intervention study in Malawi in 3 clinics managed by the DREAM program (Disease Relief through Excellence and Advanced Means) in Kapire, Kapeni and Balaka. Each clinic piloted a different intervention: Kapire, the use of male champions; in Balaka, the introduction of a special day for male patients; in Kapeni incentives (nudges; a food package) for male partners attending the clinic. The study protocol originally included a control center where no intervention was performed. This center was later removed from the study as we encountered some technical problems that affected the collection of data.

DREAM is a health program run by the Community of Sant'Egidio aimed at addressing a wide range of health challenges according to the needs of different contexts (37–40). The program is now operating in 10 African countries in several sectors (HIV care, TB treatment, cervical cancer screening and prevention, hypertension and diabetes control, neurological disorders treatment), and its peculiarity is the holistic and personalized approach adopted by the healthcare workers and staff.

The paper is the first output of a pilot project, called “WeMen,” that aims to evaluate the impact of three different interventions on adherence and the retention to Antiretroviral therapy (ART) of HIV-positive women (36). The methodology and protocol of the study were extensively presented elsewhere (36), and the study's protocol was registered at ClinicalTrials.gov (ID: NCT04356157 URL: https://clinicaltrials.gov/ct2/show/NCT04356157).

The baseline analysis was conducted from July 2019 to March 2020; the interventions took place from August 2020 to January 2021 and the post-intervention from October 2020 to March 2021. We included HIV+ women in both the pre-and post-intervention periods who agreed to join the study. Women younger than 18 years and with no stable partner were excluded.

In the healthcare centers where we carried out the interventions, we asked women to invite their male partners to the health center for HIV testing and counseling, and we administered a questionnaire to the woman at the subsequent visit (Supplementary File 1). The questionnaire explored various dimensions related to partner support and sexual and reproductive health, including the level of support each woman received from their partner in terms of accompaniment or emotional support, their satisfaction, and any harms the women faced within the household, as detailed described in the protocol. The final questionnaire consisted of 29 questions. A selection of questions is reported in Box 1. Trained medical doctors and clinical officers administered this questionnaire before and after the interventions in all the health centers. Through this survey, we evaluated the impact of the interventions on the male partner involvement in each facility adopting the following intermediate outcomes: (i) the number of women accompanied by male partners after invitation, (ii) number of men accepting to be tested for HIV, (iii) perceived partner involvement and (iv) presence of gender-based violence in the family.

Box 1 **Questions included in the baseline/post-intervention surveys and analyzed for intermediate outcomes.**

*Main outcomes*

Did your partner accept to come with you to the clinic (Yes/No)

Has your partner accepted to be tested for HIV? (Yes/No)

In general, how satisfied are you with the overall support you get from your partner? (Very dissatisfied/dissatisfied/neither/satisfied/very satisfied)

Do/did you generally feel physically or emotionally unsafe in your relationship with your partner? (Yes/No/Sometimes/I prefer not to respond)

*Other outcomes (Yes/No)*

Does your partner approve of using a contraceptive method to prevent pregnancy?

Does your partner agree that you attended antenatal clinics when you were/are pregnant?

Does your partner support transportation cost for you to get to the healthcare center/PMTCT clinic?

Did your partner help you remember to take your HIV medication during the past year?

As described in the protocol paper, the primary outcomes evaluated in the WeMen project are women's retention in care and adherence to treatment at 6 months, 1 and 2 years. These are still under evaluation and will be published in a following paper.

### Statistical Analysis

We performed statistical analysis with software “R” version 4.1.2 (41) [packages tidyverse (42), gtsummary (43), officer (44)]. The first intermediate outcome (i) is measured by the proportion of affirmative responses to the question “*Did your partner accept to come with you to the clinic?”* over the number of women included in the study. The second outcome (ii) is calculated as the proportion of affirmative responses to the question “*Has your partner accepted to be tested for HIV?”* over the number of women whose partner agreed to accompany her to the clinic. The third outcome (iii) was measured with a Likert scale with values from 1 (very dissatisfied) to 5 (very satisfied) to the question “*In general, how satisfied are you with the overall support you get from your partner?”* The fourth intermediate outcome (iv) was measured by the proportion of negative responses to the question “Do/did you generally feel physically or emotionally unsafe in your relationship with your partner?” over the total number of women included in the study. Other outcomes related to male involvement were measured with dichotomic questions where the affirmative response indicated a positive attitude from the male partner. The questions related to the four “main outcomes” and the 5 “other outcomes” are reported in Box 1. The full survey with all questions is attached as Supplementary File 1.

The same data were collected in the pre-and post-analysis using specific software, respecting patient privacy and data security rules. We used a two-sample test of proportions to test the difference between outcomes expressed by rates at baseline and post-intervention, and we calculated a 95% confidence interval for differences in proportions. We used the Kruskal-Wallis test to test the difference in the distribution of responses to indicator three expressed with a Likert scale.

The study protocol has been reviewed and approved by the College of Medicine Research and Ethics Committee (COMREC) under Malawi's National Health Science Research Committee (NHSRC) with approval number 2021.

## Results

### Baseline and Post-intervention Analysis

In total, 324 women were included at the baseline and 381 in the post-intervention evaluation, as shown in Table 1.

**Table 1:** Surveys delivered per center at baseline and post-intervention.

**Center**	**Baseline**	**Post_intervention**
Male champions (Kapire)	75	84
Nudge (Kapeni)	78	109
Special day (Balaka)	171	188
Total	324	381

As reported in Tables 2, 345 men participated in the “Special Day” intervention; 137 couples were eligible for the “Nudge” intervention, and among these 91 men came to the center with their partner when we offered a food package; the “Male champions” intervention reached 1,488 men with a total of 72 meetings in the communities. Table 2 shows the participation in interventions stratified per month, and regarding the “Special Day” intervention, it reports the number of patients accessing health services. At the end of the “Special Day”, among 345 participants, 36% of them were satisfied with the service, and 64% were very satisfied. Table 3 reports the details and the socio-demographic data of participants to this intervention.

**Table 2:** Indicators on the delivery of the interventions.

**Intervention**	**Indicator**	**August**	**September**	**October**	**November**	**December**	**January**	**Total**
Special days (Balaka)	Participants	43	42	84	107	41	28	345
	Number of VCTs (% of participants)	3 (7.0%)	11 (26.2%)	17 (20.2%)	11 (10.3%)	5 (12.2%)	3 (10.7%)	50 (14.5%)
	Number of nutritional counseling (% of participants)	6 (14.0%)	23 (54.8%)	0 (0.0%)	22 (20.6%)	0 (0.0%)	0 (0.0%)	51 (14.8%)
	Number of glucose screenings (% of participants)	5 (11.6%)	25 (59.5%)	35 (41.7%)	0 (0.0%)	12 (29.3%)	0 (0.0%)	77 (22.3%)
	Number of BP tests (% of participants)	10 (23.3%)	26 (61.9%)	50 (59.5%)	28 (26.2%)	0 (0.0%)	6 (21.4%)	120 (34.8%)
Nudge (Kapeni)	Eligible couples	9	34	17	13	33	40	137
	Incentives delivered (% of eligible couples)	3 (33.3%)	34 (100.0%)	5 (29.4%)	12 (92.3%)	21 (63.6%)	19 (47.5%)	91 (66.4%)
Male champions (Kapire)	Meetings organized	13	15	13	14	16	14	72
	Total participants	223	369	251	291	323	254	1,488

**Table 3:** Special day participants characteristics (Balaka).

**Characteristic**	***N*** **= 345^a^**
Age of participants	33 (25, 47)
**Level of satisfaction**	
Not satisfied	2 (0.6%)
Satisfied	123 (36%)
Very satisfied	220 (64%)
Participant living with a female partner	261 (76%)
**Profession**	
Employee	57 (17%)
Small business	53 (15%)
Farmer	74 (21%)
Piece work	66 (19%)
Unemployed	22 (6.4%)
Other	73 (21%)

a *Median (IQR); n (%)*.

### Intermediate Outcomes

**Figure 1:**
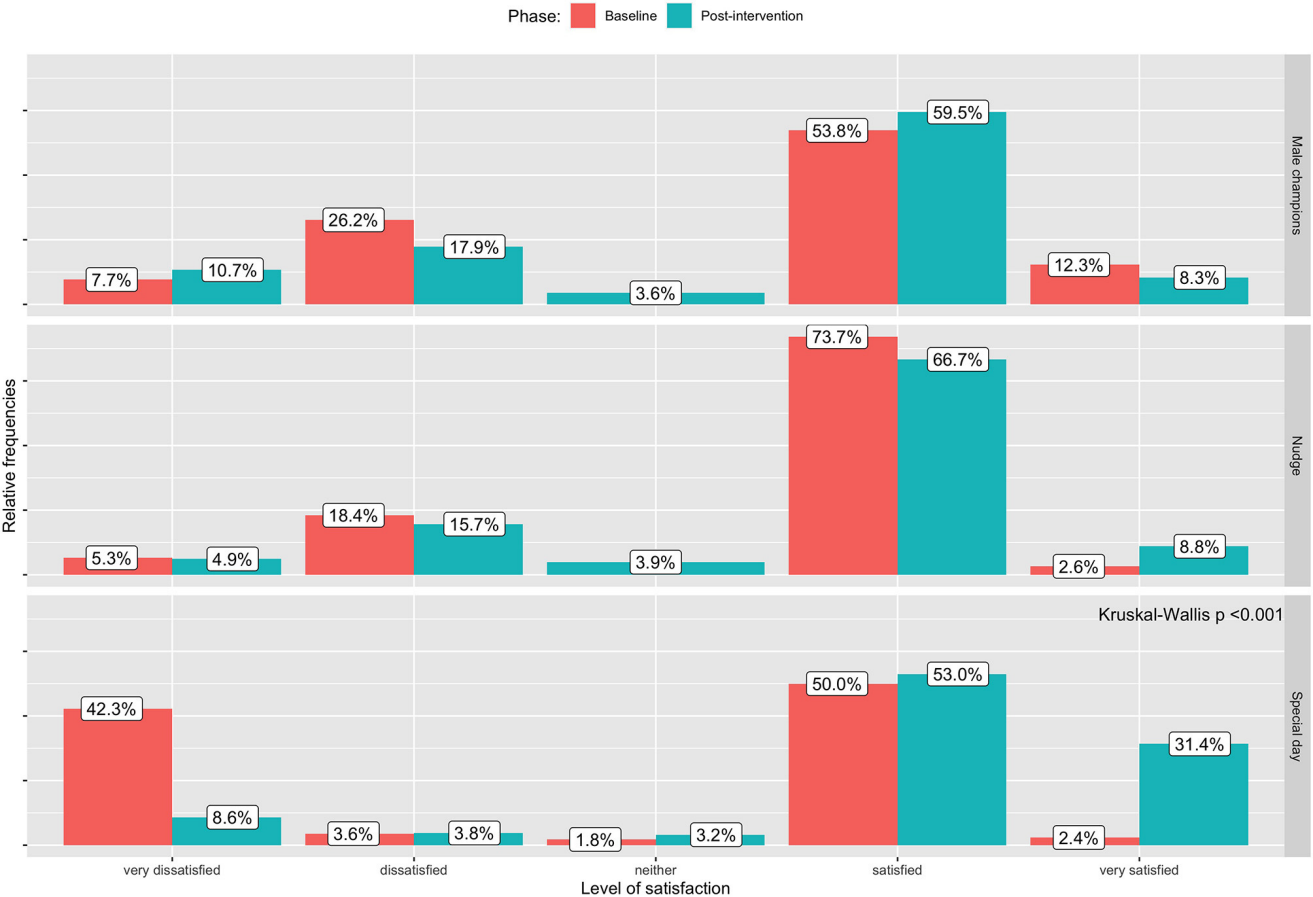
Impact of the interventions on the intermediate outcome (iii): level of satisfaction expressed by women on the support that they are receiving by their male partners at baseline and post-intervention.

Table 4 and Figure 1 show the intermediate outcomes at baseline and post-intervention by the center. Table 4 reports the proportion of women accompanied by their respective partners to the facility after receiving the invitation card, the proportion of men who accepted the HIV test and counseling and the level of domestic violence as perceived by the woman (main outcomes), plus the responses of women to some questions used to evaluate the male support with respect to health issues (other outcomes). The outcome on the level of satisfaction expressed by the woman concerning the support of the male partner evaluated with a Likert scale is shown in Figure 1.

**Table 4:** Intermediate outcomes at baseline and post-intervention.

	**Indicator**	**Intervention**	**Baseline**	**Post-intervention**	**Difference**	***P*** **-value**	**C.I. low**	**C.I. high**
Main indicators	Proportion of women accompanied	Special day^**^	48.5% (83/171)	81.4% (153/188)	32.8%	<0.001	0.23	0.43
		Nudge	53.8% (42/78)	67.9% (74/109)	14.0%	0.07	−0.01	0.29
		Male champions	84.0% (63/75)	84.5% (71/84)	0.5%	1.00	−0.11	0.12
	Proportion of men accepting testing over those accompanying partner	Special day^**^	86.7% (72/83)	65.4% (100/153)	−21.4%	<0.001	−0.33	−0.10
		Nudge	95.2% (40/42)	98.6% (73/74)	3.4%	0.61	−0.05	0.12
		Male champions	88.9% (56/63)	95.8% (68/71)	6.9%	0.24	−0.04	0.17
	Proportion of women feeling safe at home	Special day^**^	63.2% (108/171)	95.2% (179/188)	32.1%	<0.001	0.24	0.40
		Nudge	83.3% (65/78)	80.7% (88/109)	−2.6%	0.79	−0.15	0.10
		Male champions^**^	44.0% (33/75)	75.0% (63/84)	31.0%	<0.001	0.15	0.47
Other indicators	Proportion of partners approving contraceptive method	Special day	80.7% (138/171)	83.5% (157/188)	2.8%	0.58	−0.06	0.11
		Nudge	64.1% (50/78)	61.5% (67/109)	−2.6%	0.83	−0.18	0.12
		Male champions	69.3% (52/75)	83.3% (70/84)	14.0%	0.06	−0.00	0.28
	Proportion of partners approving healthcare access	Special day	84.2% (144/171)	91.0% (171/188)	6.7%	0.07	−0.01	0.14
		Nudge	61.5% (48/78)	61.5% (67/109)	−0.1%	1.00	−0.14	0.14
		Male champions^*^	78.7% (59/75)	94.0% (79/84)	15.4%	0.009	0.04	0.27
	Proportion of partners approving antenatal visits	Special day	83.0% (142/171)	88.3% (166/188)	5.3%	0.20	−0.03	0.13
		Nudge	65.4% (51/78)	63.3% (69/109)	−2.1%	0.89	−0.17	0.13
		Male champions^*^	65.3% (49/75)	85.7% (72/84)	20.4%	0.005	0.06	0.35
	Proportion of partners supporting with transportation cost	Special day	81.3% (139/171)	87.8% (165/188)	6.5%	0.12	−0.02	0.15
		Nudge	57.7% (45/78)	50.5% (55/109)	−7.2%	0.41	−0.23	0.08
		Male champions	53.3% (40/75)	61.9% (52/84)	8.6%	0.35	−0.08	0.25
	Proportion of partners supporting reminding drugs prescription	Special day	81.3% (139/171)	87.8% (165/188)	6.5%	0.12	−0.02	0.15
		Nudge	57.7% (45/78)	50.5% (55/109)	−7.2%	0.41	−0.23	0.08
		Male champions	53.3% (40/75)	61.9% (52/84)	8.6%	0.35	−0.08	0.25

* *p-value < 0.01;*

** *p-value < 0.001*.

### Special Day (Balaka)

In the site where the special day intervention was implemented, we observed a significant increase in male involvement from baseline to post-intervention in terms of the number of women accompanied from their partners after delivering an invitation card (from 48.5 to 81.4%; *p* < 0.001), the number of women feeling safe at home (from 63.5 to 95.2%; *p* < 0.001) and the level of satisfaction of female partners about male support (proportion of satisfied and very satisfied from 52.4 to 84.4%; *p* < 0.001). On the other hand, the proportion of men who accepted Voluntary Counseling and Testing (VCT) when they accompanied their female partner to the clinic decreased (from 86.7 to 64.4%).

### Male Champions (Kapire)

In the center where the male champions intervention was implemented, an increment was observed in the proportion of women feeling safe at home (from 44.0 to 75%; *p* < 0.001) and in some other outcomes, such as the proportion of men approving the access of their partners to healthcare services (from 78.7 to 94.0%; *p* = 0.009) and antenatal care (from 65.3 to 85.7%; *p* = 0.005).

### Nudge (Kapeni)

No significant difference in male involvement between the baseline and the post-intervention was reported in the site implementing the “Nudge” intervention.

## Discussion

The study showed that each intervention enhanced the proportion of women accompanied by their male partners to the facility. However, this increment significantly increased only after implementing the special day for men in Balaka. Women felt safer at home after the special day and male champions intervention. Moreover, the male champions may have improved the proportion of partners supporting the women's access to the clinics and antenatal visits.

Previous literature reviews on approaches adopted to enhance male support reported that psychosocial interventions and complex community interventions (or multi-component) are promising interventions in the uptake of maternal antiretroviral therapy and male support (34, 35). Complex community interventions usually reported an effect on multiple levels of the health behavior model; several approaches, such as the adoption of male champions (45, 46), the adoption of the male-friendly clinic (30, 46), the use of drama and talks during daily life (47, 48), and the adoption of household couples' support (49) showed a substantial effect on male involvement. In a pre-and post-intervention study in 116 antenatal clinics in Kenya where a package of interventions (including health education activities in the facilities, the delivery of the invitation letters to the women, and adoption of peer-to-peer educators) has been delivered, the number of men accompanied by their female partners increased from 7.4% at the baseline to 54.2% 3 months after the intervention (RD = 0.47, CI 0.45–0.48) (50). Our findings show that multi-component interventions such as adopting male champions that roll out educational activities within the communities and opening the facilities for men providing targeted clinical and education services for men may be helpful to promote male partner support. Moreover, our findings showed a higher proportion of men accompanying their female partner to the facilities (above 67%) than most previous interventions in Malawi and SSA (25–30, 32, 33). This result may be explained by several factors, such as the gender-sensitive approach adopted throughout the interventions and the increasing number of interventions and increasing attention on the male engagement within the country (51).

However, since these are multi-component interventions, it is difficult to attribute the effect of an intervention to one component both in our study and in previous studies where the effect of every single component was not reported (34, 52).

### Limitations

Some limitations of this study are related to the design adopted and the local context, and we have reported them in the published protocol of the study (36). These include a selection bias linked to the non-randomized assignment of the interventions and the setting of the interventions, such as a health program offered by a non-governmental organization that follows non-standard protocols aiming at a higher quality of services. Furthermore, the data available to us does not allow us to take into account any confounders that may have influenced the results.

In order to produce more reliable results, it would have been necessary to include a more significant number of facilities, randomize the selection of facilities, calculate a sample size capable of limiting systematic errors, and prolonging both the enrollment period and the duration of the interventions tested. However, these activities would have entailed unaffordable costs, and longer study duration was not allowed in this research project. Furthermore, during the study, difficulties emerged in data collection caused by a high workload and a high turnover for the health personnel involved, also because of the COVID-19 outbreak after the start of the study. Due to these difficulties, it was impossible to collect data of sufficient quality in the control center that would have allowed us to assess whether the results derived from temporal trends or unmeasured events.

In addition to these limits, a social desirability response bias may be a confounder and lead to inaccurate self-report conclusion: some women may have had privacy concerns; they may not want to reveal unsupportive behaviors of their male partner since they do not want to endanger their relationship and preferred to preserve their relationship avoiding possible interferences and conflicts within the couple (53). These biases may have skewed the results toward an overestimation of male involvement and, therefore, underestimating the effectiveness of interventions aimed at increasing it. To minimize this bias, the team used the expertise of the NGO responsible for managing the centers to create a safe environment in which patients feel free to share their problems and difficulties. The main points of this approach are the use of expert clients (peer educators) and systematic training of medical staff based on the holistic approach adopted in the protocols of the centers.

One of the strengths of this study is having adopted an ecological approach with interventions aimed at individuals but targeting facilities or communities (54, 55). On the other hand, this approach does not assess whether the intervention causes the behavior change explicitly as the context mediates the effect. For example, although the interventions took place in small localities such as villages, it is not sure that the men involved in the educational activities were the partners of the women on whom the effect was measured or that the men themselves had relationships with the women's partners.

A further limitation of the study that limits its reproducibility and comparison with other studies on the subject is the absence of a validated standard tool for measuring male involvement. However, using several indicators allows us to interpret the results from multiple points of view and compare them with studies that have used the same indicators.

### Implications

Our results have important implications from a public health perspective that support the feasibility and effectiveness of specific interventions aimed at male involvement. As a general consideration, our findings highlight the need for a comprehensive intervention aimed at a multi-level impact (ecological model) on the social context related to the male-female interaction. In our study, *Special days* and *Male champions* were the interventions able to consistently increment the studied variables, while nudges were feebly impacting male involvement; this could mean that the social and context aspects, rather than the individual/material ones, should be considered when planning specific programs to increase male involvement. The management of health care units and the design of services is strongly implicated in male partners' possible involvement. Hence, the delivery of male-friendly services should be considered a responsibility of the policymakers and service providers, both at the local and national levels. Further studies could be helpful to evaluate more in detail some aspects. In particular, the possible effect of confounders factors not considered in our analysis (age of participants, rural/urban context, socio-cultural level of households, and others), the specific aspects of the special days' intervention that could be more effective in the male involvement increment, and others.

However, in our opinion, the study can significantly contribute to the design of more male-friendly services to enhance both male- and female-health status. Local and international guidelines on HIV care should consider more clearly MI and include specific interventions aimed at its improvement.

## Conclusions

Complex public health interventions acting on multiple levels could effectively increase the male involvement in the health of their female partners, as reported by the same women. In particular, the realization of special opening days of clinics reserved for men with health education services and awareness campaigns in the communities with male champions proved to be the most promising interventions. The ecological approach used in these interventions, which also act on the environment/context and not only on individuals, and having acted both at the clinical facility level and at community and social network level, are the aspects that probably have increased the effectiveness of the interventions. However, the ecological approach does not elicit the direct causal mechanisms and the effectiveness of the individual components of the interventions. Moreover, it is necessary to adopt standard and validated criteria to measure male involvement in health services. It is recommended to replicate interventions of this type in other contexts to produce more evidence of their effectiveness and observe the impact on final health outcomes such as adherence and retention in care in programs for the treatment of chronic diseases. Given the limitations of the context and the availability of time and resources, the results should be interpreted with caution. They should be intended as a first step in order to test the most promising interventions in more extensive studies to verify their validity.

## Data Availability Statement

The raw data supporting the conclusions of this article will be made available by the authors, without undue reservation.

## Ethics Statement

The studies involving human participants were reviewed and approved by College of Medicine Research and Ethics Committee (COMREC) under Malawi's National Health Science Research Committee (NHSRC) with approval number 2021. The patients/participants provided their written informed consent to participate in this study.

## Author Contributions

IT, FC, IP, and SO: conceptualization, methodology, and writing—original draft. BM and DT: data curation. BM and SO: formal analysis. SO: funding acquisition and visualization. IT and SO: investigation. FC: project administration. FC and SO: resources. LP and SO: supervision. MM, LP, and GT: validation and writing—review & editing. All authors have read and agreed to the published version of the manuscript.

## Funding

This research was funded by the Italian Ministry of Foreign Affairs through the Italian Agency for International Cooperation within the Global Fund 5% program aimed at providing evidence on how to improve the interventions of the Global Fund against tuberculosis, HIV, and malaria in the countries where the fund operates. Grant WeMen! Improving womens access to health care system through mens inclusion AID 011587/08/05.

## Conflict of Interest

The authors declare that the research was conducted in the absence of any commercial or financial relationships that could be construed as a potential conflict of interest.

## Publisher's Note

All claims expressed in this article are solely those of the authors and do not necessarily represent those of their affiliated organizations, or those of the publisher, the editors and the reviewers. Any product that may be evaluated in this article, or claim that may be made by its manufacturer, is not guaranteed or endorsed by the publisher.
