# Supplementary material for: Improving Male Partner Involvement in HIV-Positive Women's Care Through Behavioral Change Interventions in Malawi (WeMen Study): A Prospective, Controlled Before-and-After Study
Source: Front Public Health. 2022 Jul 8;10:864489. doi: 10.3389/fpubh.2022.864489 (PMC9305193; doi:10.3389/fpubh.2022.864489)
Supplement: Supplementary file 1 [file Table_1.DOCX]

*
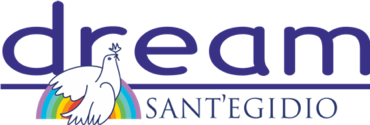

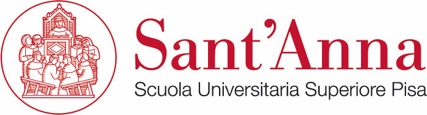

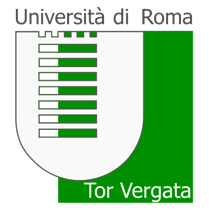
*

“WEMEN! IMPROVING WOMEN'S ACCESS TO HEALTHCARE SYSTEM

THROUGH MEN'S INCLUSION”

Questionnaire

V 3.0

**SURVEY**

**General information about the partner**

1. What is your relationship to the men living with you (your partner)?

- Husband/wife
- Boyfriend/regular partner
- Other, specify:___________

1. How long have you and your partner been living together? Months ____ or years ___
2. How many living children do you have with your partner? Number of children_____
3. How old is your partner? _____
4. What is the highest level of education that your partner has achieved?

- Primary school certificate
- Secondary school certificate
- Graduate degree
- I don’t know

1. Has your current/most recent partner had a relationship with other women while still with you?

- Yes
- No
- Maybe
- I don’t know
- I prefer not to respond

1. Has your current partner had children with any other woman while with you?

- Yes
- No
- Maybe
- I don’t know
- I prefer not to respond

**Partner’s HIV-status**

1. Did your partner go for HIV test?

- Yes
- No

*Please, respond to answer 9*

- I don’t know

8.1 If yes, is your partner HIV positive?

- Yes
- No

*Please, respond to answer 9*

- I don’t know

8.2 If yes, is your partner taking ART?

- Yes
- No

*Please, respond to answer 9*

- I don’t know

**Partner’s knowledge of healthcare services**

1. Does your partner know the service of PMTCT?

- Yes
- No
- I don’t know

**Partner’s participation in family planning and reproductive health practices**

1. Does your partner approve using a contraceptive method to prevent pregnancy?

- Yes
- No

1. Does your partner approve that you go to the healthcare center/PMTCT clinic?

- Yes
- No

1. Does your partner agree for you to attend antenatal clinics when you were/are pregnant?

- Yes
- No

1. Does your partner accompany you to the healthcare center/PMTCT?

- Yes
- No

1. Did your partner accompany you to the delivery of your last child?

- Yes
- No

1. Does your partner support transportation cost for you to get to the healthcare center/PMTCT clinic?

- Yes
- No
- Not needed because cost is irrelevant

1. During the past year, did your partner help you to remember to take your HIV medication?

- Yes
- No

1. During the past year, what has your partner done to help you remember to take your HIV medication (Check all that apply).

- remind me to take my ARVs
- attend monthly visits with me
- offer emotional support in taking drug

**Woman’s perspective about partner’s support**

1. What is your opinion of the support received from your partner? (Please choose one answer)

- I receive all the support I need
- I receive almost all the support I need
- I only receive a small amount of support
- I receive no support at all
- Other, please specify

1. In general, how satisfied are you with the overall support you get from your partner? (Likert scale: 1 very dissatisfied -5 very satisfied)

**Sample Intimate Partner Violence (IPV) Screening Questions (reference: USAID IPV and WHO 2005)**

1. Can you tell me if your current partner has ever done the following things to you? (You can choose more than one answer)

- Insulted you or made you feel bad about yourself
- Humiliated you in front of other people
- Threatened you
- Hit or hurt you in any way
- Refused to help you with transportation money to the clinic when you asked for it
- Stopped you from earning income on your own
- I prefer not to answer

1. Every couple has conflicts. What does/did your partner usually do when you have/had a disagreement? (Please choose one answer)

- We just argue
- He shouts at me
- He hits me
- He leaves me and walks away
- He refuses to provide care to the family
- I prefer not to respond

1. Do/did you generally feel physically or emotionally unsafe in your relationship with your partner?

- Yes
- No
- Sometime
- I prefer not respond

1. Comments

**QUESTIONNAIRE**

1. Have you ever come to DREAM with your partner in the past, before receiving the invitation card we just gave you?

- Yes
- No, please respond to question 2.

1.1 If yes, did your partner accept to be tested for HIV?

- Yes
- No, please respond to question 2.

1.2 If yes, did your partner test positive?

- Yes
- No, please respond to question 2.

1.3 If yes, has your partner started ART Therapy?

- Yes
- No, please respond to question 2.

1.4 If yes, is your partner accessing therapy in the DREAM program?

- Yes
- No

1.5 If yes, please tell me your partner DREAM ID _ _ _ _ _ _

1. Are you with the same partner since the time you know your status?

Yes

No

2.1 If no, was your separation related to your HIV status.

Yes

No

1. Have you delivered the invitation card to your partner?

- Yes
- No, please respond to question 3.8

3.1 If yes, did your partner accept to come with you to the clinic?

- Yes
- No

3.2 If yes, when did he come with you to the clinic approximately?

- Within 7 days after receiving the invitation card
- Within 30 days after receiving the invitation card
- More than 30 days after receiving the invitation card.

3.3 Has your partner accepted HIV testing?

- Yes
- No, please respond to question 3.8

3.4 If yes, has your partner tested positive?

- Yes
- No

3.5 If yes, has your partner started ART Therapy?

- Yes
- No

3.6 If yes, is your partner accessing therapy in the DREAM program?

- Yes
- No

3.7 If yes, please tell me your partner DREAM ID _ _ _ _ _ _

3.8 If no, why didn't you deliver the invitation card? (Please choose one answer)

- Atmosphere within the family (there are some disagreements in the family)
- Fear of partner reaction
- I don’t want his involvement because PMCTC is my responsibility
- I have not disclosed my status to my partner
- My partner goes to another center (not DREAM)
- Other, please specify the reason

**UPDATES (In case the women come for a subsequent visit during the six months of data collection)**

1. Did your partner accept to come with you to the clinic?

- Yes
- No

1. When did he come with you to the clinic?

Day/ month/ year __ __ / __ __ / __ __ __ __

1. Has your partner accepted to be tested for HIV?

- Yes
- No

3.1 If yes, has your partner tested positive?

- Yes
- No

3.2 If yes, has your partner started ART Therapy?

- Yes
- No

3.3 If yes, is your partner accessing therapy in the DREAM program?

- Yes
- No

3.4 If yes, please tell me your partner DREAM ID _ _ _ _ _ _
